# Direct Oral Anticoagulants Versus Vitamin K Antagonists for Treatment of Left Ventricular Thrombus: A Review of Efficacy and Safety

**DOI:** 10.31083/RCM50947

**Published:** 2026-08-18

**Authors:** Malik Alqawasmi, Osaid Alrahamneh, Altamash Jawadi, James C. Blankenship

**Affiliations:** ^1^Department of Internal Medicine, University of New Mexico, Albuquerque, NM 87106, USA; ^2^Division of Cardiology, Department of Internal Medicine, University of New Mexico, Albuquerque, NM 87106, USA

**Keywords:** heart ventricles, thrombosis, anticoagulants, warfarin, treatment outcome

## Abstract

Left ventricular thrombus (LVT) remains a critical complication of acute myocardial infarction (MI), particularly in patients with anterior ST-segment elevation myocardial infarction (STEMI) and apical dysfunction. While primary percutaneous coronary intervention (PCI) has reduced its overall incidence compared with the pre-thrombolytic era, LVT continues to pose a significant risk of systemic embolization and stroke, driven by the interplay of endothelial injury, hypercoagulability, and stasis within the aneurysmal apex. This review synthesizes current evidence on the pathophysiology, diagnosis, and management of LVT. We critically evaluate diagnostic modalities, highlighting the substantial sensitivity gap between the clinical standard, transthoracic echocardiography (TTE), and the gold standard, cardiac magnetic resonance (CMR). This discrepancy has important implications for clinical decision-making, as TTE often overestimates thrombus resolution, potentially leading to the premature cessation of therapy. Therapeutically, the landscape has shifted from the empiric use of vitamin K antagonists (VKAs) toward the adoption of direct oral anticoagulants (DOACs). Thus, we analyze recent randomized data, including the Comparative Study of Oral Anticoagulation in Left Ventricular Thrombi (No-LVT) and the Rivaroxaban vs Warfarin in Acute Left Ventricular Thrombus Following Myocardial Infarction (RIVAWAR) trials, which demonstrate that DOACs are non-inferior to VKAs for embolic prevention and may offer superior resolution velocity, a crucial metric for stabilizing friable thrombi. We conclude that DOACs represent a reasonable, patient-centered alternative to warfarin. Nonetheless, future management strategies must integrate precise, serial imaging with rapid-onset anticoagulation to ensure complete histologic resolution while minimizing the hemorrhagic burden of prolonged triple antithrombotic therapy.

## 1. Introduction

Left ventricular thrombus (LVT) represents a critical complication of severe left ventricular dysfunction, most commonly arising after acute myocardial infarction (MI) or in the setting of dilated cardiomyopathy with reduced ejection fraction. The pathophysiology of LVT is primarily driven by Virchow’s triad: endothelial injury, blood stasis in the ventricular apex, and a systemic hypercoagulable state, which together create a high-risk environment for clot formation. The primary clinical concern is the significant risk of systemic embolization, particularly ischemic stroke, making prompt diagnosis by transthoracic echocardiography (TTE), often with contrast, or cardiac magnetic resonance imaging (MRI) essential. Meanwhile, management focuses on systemic anticoagulation, traditionally with vitamin K antagonists (VKAs); however, emerging evidence has increasingly supported the use of direct oral anticoagulants (DOACs) to reduce embolic risk and promote thrombus resolution.

### 1.1 Definition and Epidemiology

LVT is a potentially life-threatening complication characterized by an echo-dense mass within the left ventricular (LV) cavity, typically adjacent to a region of abnormal wall motion and with margins distinct from the myocardium [1]. While the widespread adoption of early revascularization via primary percutaneous coronary intervention (PCI) has significantly reduced the prevalence compared with the pre-thrombolytic era, when incidence rates were reported as high as 46% [2,3], LVT remains a frequent clinical challenge. In the contemporary era, LVT occurs in 2% to 7% of patients after acute MI [4]. This incidence increases substantially to approximately 19% in patients presenting with anterior wall ST-segment elevation myocardial infarction (STEMI) and significantly reduced left ventricular function [4]. LVT most commonly develops after large anterior infarctions [5], in which LV ejection fraction (LVEF) falls below 40%, creating a physiological environment characterized by blood stasis, endocardial injury, and localized inflammation [1].

### 1.2 Clinical Implications

The presence of LVT carries serious prognostic significance; if left untreated, the risk of embolization is 10%–20% [6,7]. Systemic thromboembolism (STE), which most frequently presents as ischemic stroke, is the primary concern, with post-MI stroke risk estimated to be 44-fold higher within the first 30 days after the event than baseline stroke risk [8]. Meanwhile, untreated LVT is associated with a 4-fold increase in stroke or systemic embolization and a 2-fold increase in long-term mortality compared with patients without LVT [9]. In addition to the immediate risk of stroke, embolic events can involve other vascular beds, leading to acute limb ischemia or mesenteric ischemia. The risk of embolization appears highest during the first one to two weeks post-MI, as fresh, mobile, or protruding thrombi are more likely to fragment [10]. Over the following three months, the thrombus typically becomes more organized, fibrotic, and adherent to the LV wall, which may reduce the associated embolic potential; however, persistent mobile thrombi remain a long-term threat [2].

### 1.3 Historical Context

For decades, VKAs, particularly warfarin, have been the standard of care for LVT management. Guideline recommendations for VKAs were largely derived from early observational studies conducted in the pre-reperfusion era, which demonstrated the associated efficacy in reducing systemic embolization [7,11,12]. Standard therapy typically consists of dose-adjusted warfarin targeting an international normalized ratio (INR) of 2.0 to 3.0 for 3 to 6 months, or until thrombus resolution is confirmed [13]. Despite the efficacy of VKAs, this therapeutic option presents substantial clinical challenges. Meanwhile, warfarin management requires careful and frequent INR monitoring, is subject to numerous drug–drug and food–drug interactions, and possesses a narrow therapeutic window [14]. Furthermore, many patients struggle to maintain a high time in therapeutic range (TTR), and suboptimal control is directly associated with lower rates of thrombus resolution.

### 1.4 The Paradigm Shift

The limitations of VKAs have driven a significant shift toward evaluating DOACs as a viable alternative. Agents such as rivaroxaban and apixaban offer several pharmacological advantages, including fixed dosing regimens, rapid onset of action, and the absence of routine coagulation monitoring [15]. The rationale for using DOACs in LVT is further supported by the established efficacy and safety of these medications in treating atrial fibrillation (AF) and venous thromboembolism, conditions that share similar pathophysiological features of blood stasis and intracardiac thrombus formation.

Current research has begun to shift scientific equipoise. While large-scale retrospective analyses, such as the Retrospective Evaluation of DOACs and Vascular Endpoints of Left Ventricular Thrombi (RED VELVT) study [16], initially raised concerns by suggesting a higher risk of stroke or systemic embolism (SSE) with DOACs than warfarin, more recent randomized evidence has challenged these findings. Additionally, as a retrospective study, RED VELVT was also vulnerable to several potential sources of bias, including treatment crossover and the inclusion of patients with AF as a competing indication for anticoagulation. Meanwhile, landmark trials such as the Rivaroxaban vs Warfarin in Acute Left Ventricular Thrombus Following Myocardial Infarction (RIVAWAR) [17] and the Comparative Study of Oral Anticoagulation in Left Ventricular Thrombi (No-LVT) [18] have demonstrated that rivaroxaban is non-inferior to warfarin for thrombus resolution, with some evidence suggesting an advantage in “resolution velocity”, allowing complete thrombus disappearance more rapidly than warfarin within the first month of therapy. Similarly, pilot trials of apixaban have confirmed the associated safety and non-inferiority in efficacy. As clinicians move toward standardizing anticoagulation protocols, this review evaluates whether DOACs should now be considered the preferred first-line strategy for LVT management.

## 2. Pathophysiology and Thrombus Dynamics

The formation and evolution of LVT are complex processes governed by the interplay of mechanical, structural, and systemic factors. Thus, understanding these mechanisms is critical for evaluating the comparative efficacy of anticoagulation strategies, particularly when considering whether the rapid onset of action of DOACs provides a mechanistic advantage over the gradual titration required for VKAs.

### 2.1 Virchow’s Triad in the Left Ventricle

The pathogenesis of LVT is classically explained by Virchow’s triad: endothelial injury, blood stasis, and hypercoagulability [1]. In acute MI, particularly STEMI, all three elements converge to create a highly thrombogenic environment within the ventricle.

**Fig. 1.**
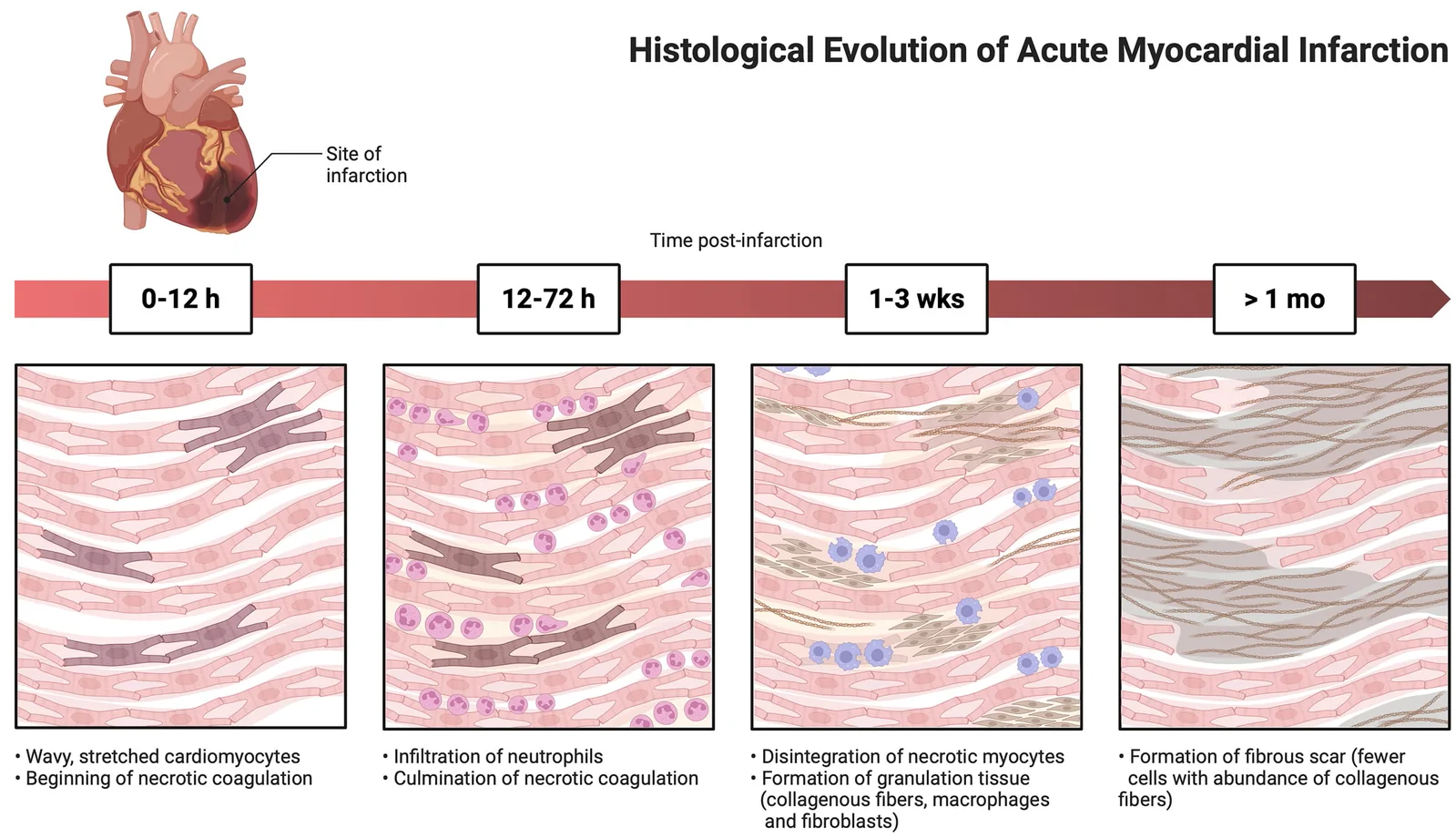
**The histological evolution of acute myocardial infarction and the subsequent deposition of subendothelial collagen**. Created in BioRender.

Endocardial injury and inflammation: The primary trigger for LVT formation is transmural infarction, which most frequently affects the anterior wall and the ventricular apex [19]. This endocardial injury exposes subendothelial collagen and tissue factor to the bloodstream (Fig. 1), thereby activating the coagulation cascade. The initial inflammatory response to myocardial necrosis further exacerbates this pro-thrombotic state. Large anterior infarctions are closely linked to this process, with most patients in modern LVT cohorts presenting with anterior STEMI and significant myocardial damage [3,13,20,21,22]. The damaged endocardium then serves as a nidus for platelet aggregation and fibrin deposition, forming the structural scaffold of the thrombus.

Hemodynamic stasis and regional wall motion abnormalities: Blood stasis is perhaps the most clinically distinct component of Virchow’s triad in this setting. The cessation of contractility in the infarcted zone, manifesting as akinesia or dyskinesia, disrupts the normal washout of blood from the ventricular apex during systole. This stagnation is particularly profound in patients with an LVEF of less than 40% [23]. The formation of an LV apical aneurysm creates a sequestered pocket of low-flow blood, allowing clotting factors to accumulate locally rather than being dispersed into the systemic circulation. Clinical imaging studies consistently reinforce this relationship; for instance, in the Apixaban in Patients With Post-Myocardial Infarction Left Ventricular Thrombus: A Randomized Clinical Trial (AP-LVT) trial [24], the persistence of LVT was significantly predicted by the presence of an LV aneurysm. The geometry of the apex, combined with impaired vortex formation during the cardiac cycle, makes this region uniquely susceptible to thrombus development. Other studies also support this relationship [25].

Hypercoagulability post-MI: While stasis and injury provide the local environment, the systemic hypercoagulable state following acute coronary syndrome (ACS) fuels thrombus growth. The acute phase of STEMI is characterized by heightened platelet reactivity and elevated levels of circulating procoagulants [1]. This systemic state helps explain why thrombus formation is most aggressive in the immediate post-infarction period, with incidence peaking within the first two weeks.

### 2.2 Thrombus Evolution

The physical character of an LV thrombus is not static; instead, an LV thrombus undergoes a dynamic evolution that directly dictates the risk of SSE [17]. This natural history is crucial for understanding why “time to resolution” has emerged as a key metric in recent trials comparing DOACs and warfarin.

In the acute phase (typically the first 1–2 weeks post-MI), the thrombus is composed of fresh, friable fibrin and platelet aggregates [26]. Morphologically, these thrombi often appear mobile or protruding into the ventricular cavity on echocardiography. This phase represents the period of highest vulnerability for the patient. The risk of embolization is estimated to be 44-fold higher within the first 30 days post-MI than in subsequent years [2,8]. Since the thrombus has not yet organized or adhered firmly to the endocardium, the thrombus is prone to fragmentation and ejection into the systemic circulation. This “fresh” state explains why early detection and rapid therapeutic anticoagulation are paramount; the goal is to dissolve or stabilize the mass before mechanical forces dislodge the mass.

Over approximately 1 to 3 months, the thrombus typically undergoes organization. Infiltration by fibroblasts and endothelial cells gradually transforms the friable mass into organized fibrotic tissue [26]. By the 3-month mark, the risk of embolization generally declines as the thrombus adheres to the aneurysmal wall, becoming a mural fixture rather than a mobile hazard. However, this transition is not guaranteed; persistent mobile thrombi can remain a threat beyond this window. The finding that thrombi are most dangerous in the early, unorganized state underscores the limitation of traditional VKA therapy, which may take days to reach therapeutic levels, potentially leaving the patient unprotected during the most critical window of thrombus fragility.

The concept of “resolution velocity”, which refers to how quickly an anticoagulant can achieve complete thrombus dissolution, has become a major focus of modern research [27,28]. Recent randomized trials suggest that DOACs may promote faster thrombus resolution than VKAs. For example, the RIVAWAR trial [17] demonstrated a significantly higher resolution rate with rivaroxaban compared with warfarin at just 4 weeks (20% vs. 8%; *p* = 0.017). This finding was echoed by the No-LVT trial [18], which reported superior 1-month resolution (71.8% vs. 47.5%). This acceleration in resolution is clinically significant because the duration of exposure to the high-risk “fresh” thrombus state is reduced. If a DOAC can resolve a thrombus within 4 weeks rather than 12, the window for a potential embolic catastrophe can be substantially reduced. Conversely, delayed resolution or sub-therapeutic anticoagulation, which is common with VKAs due to fluctuations in TTR, prolongs the presence of mobile thrombotic material and sustains the risk of stroke or SSE. Thus, the pharmacodynamic profile of DOACs, which provides immediate and stable anticoagulation without the “ramp-up” period associated with warfarin, may be better aligned with the biological imperative to ensure fresh LV thrombi are stabilized rapidly.

## 3. Diagnostic Modalities: Impact on Management

The diagnosis and serial monitoring of LVT rely heavily on non-invasive imaging. The choice of imaging modality influences the initial detection rate and fundamentally defines the endpoints of efficacy in clinical trials, as well as the duration of anticoagulation in clinical practice. A critical tension exists between the practical standard of care, TTE, and the gold standard for tissue characterization, cardiac magnetic resonance (CMR). Computed tomography (CT) data for detecting LVT are limited, and this modality has not been validated against pathology or clinical outcomes [13,29]. However, CT has potential, especially with delayed-phase imaging and CT-derived extracellular volume mapping, which markedly enhance the detection and tissue characterization of LVT [30].

### 3.1 Transthoracic Echocardiography (TTE)

TTE remains the cornerstone of LVT diagnosis and management due to the overall widespread availability, bedside portability, and cost-effectiveness [31]. For acute MI, TTE is universally indicated to assess LVEF and regional wall motion abnormalities [32]. Since LVT invariably occurs in regions of akinesia or dyskinesia, typically at the apex, the initial thrombus screening is effectively incorporated into the standard post-MI workflow.

Notably, the vast majority of evidence guiding LVT management, including recent randomized controlled trials (RCTs) comparing DOACs with VKAs, relies almost exclusively on TTE. The No-LVT trial [18], the AP-LVT trial [24], and the RIVAWAR trial [17] all used TTE as the primary imaging modality to define both baseline thrombus presence and thrombus “resolution” at follow-up. Consequently, the metric of “time to resolution” discussed in the literature is, by definition, “time to resolution on echocardiography”.

Despite the utility of standard TTE, this technique has well-documented limitations in evaluating the left ventricular apex. The apex is located in the near field of the ultrasound transducer, making it susceptible to near-field clutter and artifacts that can obscure a thrombus [33]. Furthermore, apical foreshortening, a common technical error where the imaging plane cuts through the ventricle proximal to the true apex, can lead to small apical thrombi being missed entirely.

To mitigate these limitations, the use of ultrasound-enhancing agents (UEAs), or contrast agents, is strongly recommended when apical visualization is suboptimal [34]. Contrast agents opacify the ventricular cavity, outline the endocardial border, and create a filling defect where a thrombus is present. Studies have demonstrated that contrast significantly improves sensitivity, increasing detection rates from approximately 35% with non-contrast TTE to roughly 60% with contrast [35]. However, even with contrast, TTE remains fundamentally limited by physical constraints and operator dependence, creating a significant diagnostic gap.

### 3.2 The Diagnostic Gap: Comparison of TTE With CMR

CMR is widely regarded as the gold standard for diagnosing LVT [33,36]. Unlike echocardiography, which relies on geometric shape and echogenicity, CMR utilizes tissue characterization to identify thrombi. The most specific finding is based on late gadolinium enhancement (LGE) sequences [37]. Since thrombi are avascular, these structures do not take up gadolinium contrast. Therefore, thrombi appear as distinct, dark (hypointense) masses adjacent to the bright (hyperintense) scarred myocardium and the blood pool. This “tissue validation” enables CMR to detect small, mural, or laminar thrombi that do not protrude into the cavity and are often virtually invisible on echocardiography.

The discrepancy between TTE and CMR is substantial. Meta-analyses and comparative studies, including those cited in the 2022 American Heart Association (AHA) Scientific Statement [13], indicate that TTE has a sensitivity of only 24%–33% compared with CMR. However, this sensitivity improves to nearly 60% with contrast agents [35,38]. This suggests that, in routine practice and potentially in clinical trials, up to two-thirds of small thrombi may be missed without CMR.

This diagnostic gap has critical implications for the management concept of “resolution”. When a trial reports that a patient has achieved “complete resolution” of LVT at 3 months based on TTE, the study often implies only that the thrombus is no longer visible on ultrasound. Meanwhile, if assessed by CMR, a significant proportion of these patients might still harbor residual organized thrombus. This phenomenon poses a risk of “pseudo-resolution”, in which the thrombus has either shrunk below the TTE resolution threshold or become laminar and adherent to the wall, blending in with the myocardium.

Therefore, the potential for TTE to overestimate resolution creates a safety dilemma. The current standard of care, treatment with anticoagulation agents for 3 to 6 months or until resolution”, relies on the premise that imaging confirmation of resolution signals the end of embolic risk. If TTE declares a thrombus “resolved” while thrombotic material remains present, as detectable by CMR, clinicians may discontinue anticoagulation prematurely, leaving the patient vulnerable to late embolic events.

If DOACs are associated with “faster resolution” on TTE, it is theoretically possible that these agents may simply modify the thrombus morphology, making the thrombus smaller or flatter, more rapidly than warfarin, rather than achieving complete histologic clearance. Nevertheless, despite the superior sensitivity of CMR, TTE remains the pragmatic choice for serial monitoring because repeated CMR scans are logistically and financially prohibitive. Moreover, whether the higher sensitivity of CMR compared with TTE translates into better patient outcomes remains unclear [39]. Thus, the management of LVT requires a clinical synthesis: recognizing that a “negative” TTE at 3 months is a reassuring but imperfect marker of true anatomic resolution.

Notably, CMR imaging can be used when TTE raises suspicion but is not definitive, or when there is a high level of concern for LVT that is not detected on TTE [13].

## 4. The Role of Vitamin K Antagonists

### 4.1 Guidelines and Historical Standards

For nearly half a century, VKAs, particularly warfarin, have served as the pharmacological cornerstone for the treatment of LVT [40]. This historical dominance is not merely a function of longevity; rather, the dominance reflects the unique physiological environment of the left ventricle post-infarction. Unlike the venous system, the primary site of deep vein thrombosis (DVT) or the left atrial appendage, the main focus in AF, the left ventricle post-STEMI represents a complex intersection of endocardial injury, severe blood stasis, and a hypercoagulable state. Historically, the robust, multi-pathway inhibition provided by VKAs was considered the only sufficient defense against the high embolic risk associated with these stagnant red thrombi.

The establishment of warfarin as the first-line therapy was reinforced by decades of observational data and small-scale trials demonstrating the associated efficacy in reducing SSE events. Until very recently, major cardiovascular guidelines, including those from the AHA, the American College of Cardiology (ACC), and the European Society of Cardiology (ESC), unanimously recommended VKAs with a target INR of 2.0 to 3.0 for a duration of 3 to 6 months [1,13,32,41]. These recommendations were based on the premise that the degree of anticoagulation required to dissolve a structured apical thrombus necessitated the predictable, albeit cumbersome, suppression of factors II, VII, IX, and X.

Furthermore, the “traditional” status of warfarin persists because it was the only agent used in the foundational studies that defined the natural history of LVT. When clinicians discuss “resolution rates” or “embolic risk windows”, they are historically referencing data derived from VKA-treated cohorts. This has created a “legacy effect” in clinical practice; despite the emergence of newer agents, many practitioners remain reluctant to deviate from the VKA standard due to the perceived severity of the complication. The risk of a devastating embolic stroke leaves little room for what may be perceived as pharmacological experimentation. Even in the 2022 AHA Scientific Statement [13], while DOACs are finally acknowledged as a reasonable alternative, the document notes that VKAs remain the therapy with the longest track record of proven efficacy in this specific setting.

### 4.2 Challenges of VKA Therapy: Monitoring Burdens, Dietary Interactions, and the Clinical Impact of TTR

While warfarin is undeniably effective when managed optimally, the “real-world” application of warfarin in the post-MI population is associated with significant clinical and logistical challenges. The transition from a hospital setting to home care for a post-STEMI patient is a high-risk period marked by polypharmacy and frequent medication adjustments. In this context, the narrow therapeutic window of VKAs becomes a primary limitation.

The efficacy of VKA therapy is closely tied to the TTR, the percentage of time the INR of a patient remains between 2.0 and 3.0. Subtherapeutic INRs (<2.0) leave patients vulnerable to thrombus propagation and SSE, whereas supratherapeutic INRs (>3.0) significantly increase the risk of hemorrhagic transformation of an embolic stroke or gastrointestinal bleeding. Modern registry data suggest that even in well-controlled environments, many patients with LVT achieve a TTR of less than 60% [2]. This instability is particularly concerning during the first 4 weeks post-MI, the window identified by the RIVAWAR trial [17] as the period of highest resolution velocity. If a patient spends a significant portion of the first month in a subtherapeutic range due to the slow onset of warfarin or frequent dose adjustments, the rate of thrombus resolution may stall, potentially extending the period of embolic risk.

Patients after MI are often started on a combination of new medications, including high-intensity statins, beta-blockers, angiotensin-converting enzyme inhibitors, and, most importantly, dual antiplatelet therapy (DAPT). Warfarin interacts with a vast array of these agents through the cytochrome P450 system, leading to unpredictable fluctuations in anticoagulation intensity [42]. Furthermore, the need for a consistent intake of vitamin K-rich foods, such as leafy greens, can be difficult for patients to maintain during the recovery phase, leading to a “seesaw” effect in INR levels that necessitates frequent and burdensome blood testing.

Perhaps the most significant challenge in the modern era is the interaction between VKAs and antiplatelet agents. Nearly all patients with LVT require DAPT, consisting of aspirin and a P2Y12 inhibitor, for the recently stented coronary arteries. Combining a VKA with DAPT creates triple therapy, which is associated with a 2- to 3-fold increase in major bleeding compared with dual therapy [43]. This safety concern has been a primary driver in the search for alternatives. The logistical “monitoring burden”, which often requires older or debilitated post-MI patients to attend weekly or bi-weekly laboratory testing, may also reduce adherence, thereby contributing to apparent treatment failure. Collectively, these factors have transformed the traditional gold standard into a substantial clinical hurdle, setting the stage for the rapid adoption of DOACs in contemporary practice.

## 5. DOACs in LVT: Review of Randomized Evidence

The transition from VKAs to DOACs for the treatment of LVT represents the most significant paradigm shift in this field in decades. For years, the use of DOACs was strictly off-label and driven by extrapolation from AF and DVT data. However, the unique pathophysiology of LVT, including the high-flow, high-shear environment within a dyskinetic ventricle, required dedicated evidence. Over the last five years, a series of RCTs has systematically challenged the dogma that warfarin is the only effective agent, culminating in high-quality data that demonstrate non-inferiority and suggest potential superiority on specific metrics, such as resolution velocity. Table 1 (Ref. [2,16,17,18,24,44,45,46,47,48,49,50,51,52,53,54]) summarizes the RCTs, observational studies, and systematic reviews/meta-analyses relevant to this topic.

**Table 1.** **Summary of the studies that compare VKAs and DOACs**.

Trial/study name	Design	Country/region	Population (n)	Anticoagulant dose	Primary outcome	Key results
No-LVT [18]	Prospective, open-label, multicenter, randomized clinical trial	Egypt and Bulgaria	79 patients	Rivaroxaban 20 mg daily vs. dose-adjusted warfarin (international normalized ratio (INR) 2–3)	Complete left ventricular thrombus (LVT) resolution as assessed by transthoracic echocardiography (TTE) at 1, 3, and 6 months	Rivaroxaban was non-inferior, with faster resolution at 1 month (71.79% vs. 47.5%; *p* = 0.03) and comparable resolution at 6 months (87.17% vs. 80.0%).
Apixaban versus Warfarin in Patients with Left Ventricular Thrombus [2]	Prospective, multicenter, randomized, open-label clinical trial	Israel	35 patients	Apixaban 5 mg twice daily (or 2.5 mg twice daily based on criteria) vs. dose-adjusted warfarin (INR 2.0–3.0)	Presence and size of LVT 3 months after initiation of anticoagulation	Apixaban was non-inferior to warfarin, with complete resolution in 94% (16/17) of apixaban-treated patients vs. 93% (14/15) of warfarin-treated patients at 3 months (*p* = 0.026 for non-inferiority).
REWARF-STEMI [45]	Open-label, randomized pilot clinical trial and meta-analysis	Iran (Tehran)	50 patients	Rivaroxaban 15 mg daily vs. dose-adjusted warfarin (INR 2.0–2.5)	Complete LVT resolution at 3 months via non-contrast two-dimensional (2D) TTE	Rivaroxaban showed a numerically higher but not statistically significant resolution rate (76.0% vs. 54.2%; *p* = 0.12), while a meta-analysis showed less major bleeding with DOACs (95% CI: –0.12 to 0.00; *p *= 0.05).
Mansouri et al. [44]	Randomized, controlled, interventional, open-label non-inferiority trial	Iran (Isfahan)	52 post-PCI patients	Rivaroxaban 20 mg daily vs. dose-adjusted warfarin (INR 2–3)	Thrombus resolution by TTE after a 3-month follow-up	Rivaroxaban was non-inferior to warfarin, with a resolution rate of 76.9% vs. 69.2% (*p* = 0.532), and there was no significant difference in bleeding or rehospitalization.
RIVAWAR Trial [17]	Open-label, non-inferiority, randomized controlled trial	Pakistan (Karachi)	261 patients	Rivaroxaban 20 mg daily vs. warfarin (target INR 2–3)	Complete LVT resolution at 12 weeks	Rivaroxaban was non-inferior to warfarin (95.8% vs. 96.6% resolution; *p* > 0.999) and showed significantly faster resolution at 4 weeks (20% vs. 8%; *p* = 0.017).
Apixaban versus Warfarin in Patients with Left Ventricular Thrombus [46]	Pilot, prospective, single-center, randomized, single-blinded outcome study	Malaysia	27 patients	Apixaban 5 mg twice daily (or 2.5 mg twice daily) vs. warfarin (target INR 2–3)	Percentage of reduction or total resolution during the first 12 weeks	Apixaban showed effectiveness similar to warfarin; mean size reduction at 12 weeks was 65.1% (apixaban) vs. 61.5% (warfarin) (*p* = 0.816).
Apixaban in Patients With Post-Myocardial Infarction Left Ventricular Thrombus [24]	Open-label, randomized, non-inferiority clinical trial	Egypt and Saudi Arabia	50 patients	Apixaban 5 mg twice daily vs. dose-adjusted warfarin (target INR 2–3)	Complete LVT resolution at 3 months via 2D transthoracic echocardiography	Apixaban was non-inferior to warfarin (*p* < 0.036 for non-inferiority), with complete resolution at 3 months in 76% (19/25) of patients receiving apixaban vs. 80% (20/25) of patients receiving warfarin.
TriNetX LV Thrombus Study [47]	Retrospective cohort analysis using propensity score matching	United States (TriNetX research network)	2488 eligible patients (945 matched pairs analyzed)	DOACs (74% apixaban 5 mg twice daily; 26% rivaroxaban 20 mg once daily) vs. warfarin	Composite of all-cause mortality and stroke/transient ischemic attack (TIA) at 1 year	DOACs and warfarin demonstrated comparable effectiveness and safety; the 1-year composite outcome occurred in 23.8% (DOACs) vs. 26.7% (warfarin) (hazard ratio (HR): 0.93; *p* = 0.46), with similar thrombus resolution rates (~81%).
Meta-Analysis of Randomized Clinical Trials [48]	Meta-analysis of 7 randomized controlled trials (RCTs)	International (studies primarily from Asian and African centers)	564 patients (323 direct oral anticoagulants (DOACs), 241 warfarin)	Rivaroxaban (k = 4) or apixaban (k = 3) vs. warfarin	LVT resolution and stroke	No significant differences were observed between DOACs and warfarin in thrombus resolution (relative risk (RR): 1.01; *p* = 0.72), stroke (RR: 0.60; *p* = 0.64), or major bleeding (RR: 0.49; *p* = 0.20).
RED VELVT [16]	Multicenter retrospective cohort study	United States (3 academic medical centers)	514 patients	DOACs (apixaban, rivaroxaban, or dabigatran) vs. warfarin	Clinically apparent stroke or systemic embolism (SSE)	DOAC use was associated with a significantly higher risk of SSE than warfarin (adjusted HR: 2.64; 95% confidence interval (CI): 1.28–5.43; *p* = 0.008) despite similar rates of thrombus resolution.
Direct oral anticoagulants compared to vitamin K antagonist for the management of left ventricular thrombus [49]	Retrospective, observational cohort study	United Kingdom (Leeds)	84 patients (22 DOACs, 62 vitamin K antagonists (VKAs))	DOACs (rivaroxaban 20 mg, apixaban 5 mg, or dabigatran 150 mg) vs. warfarin (INR 2–3)	Rates of thromboembolism and clinically significant bleeding	No statistically significant differences were observed in stroke (0% (DOACs) vs 2% (VKAs); *p* = 0.55), bleeding (0% (DOACs) vs 10% (VKAs); *p* = 0.13), or thrombus resolution (65% (DOACs) vs 76% (VKAs); *p* = 0.33).
Management of Left Ventricular Thrombi with Direct Oral Anticoagulants [50]	Observational retrospective study	France	59 patients	DOACs vs. VKA (target INR 2–3; increased to 3–4 for non-responders)	Rate of LVT resolution	Similar resolution rates were observed (70.6% (DOACs) vs 71.4% (VKAs); *p* = 0.9). Notably, all patients (5/5) who failed with DOAC treatment achieved resolution after switching to VKA.
Gogos et al. [51]	Systematic review and meta-analysis	Various (meta-analysis)	605 post-acute myocardial infarction (AMI) patients from 8 studies	DOACs (predominantly apixaban and rivaroxaban) vs. VKAs	LVT resolution, SSE, and bleeding	DOACs demonstrated a nearly twofold higher likelihood of thrombus resolution (odds ratio (OR), 1.95), a 70% lower risk of systemic embolism (OR, 0.30), and a 54% lower risk of bleeding (OR, 0.46) compared with VKAs.
Bass et al. [52]	Retrospective cohort study	United States	949 patients (180 DOAC, 769 warfarin)	DOAC (apixaban, dabigatran, edoxaban, or rivaroxaban) vs. warfarin	Incidence of new onset thromboembolic stroke within 90 days	No differences were observed in new-onset thromboembolic stroke (7.8% (DOAC) vs. 11.7% (VKA); *p* = 0.13), composite thromboembolic events, or GUSTO bleeding. Significantly more patients receiving warfarin required blood products (25.8% vs. 13.9%; *p* < 0.001).
Huang et al. [53]	Systematic review and meta-analysis	International (meta-analysis)	3172 patients (888 DOACs, 2284 VKAs) across 21 studies	DOACs vs. VKAs	Safety and efficacy outcomes (mortality, bleeding, stroke, SE, LVT resolution)	DOACs significantly reduced the risk of bleeding events (RR 0.73; *p* = 0.004) and stroke (RR 0.72; *p* = 0.03) compared with VKAs. DOACs were also associated with higher LVT resolution at 1 month (RR 1.96; *p* = 0.008). No significant differences were noted in overall mortality or systemic embolism.
Shah et al. (ELECTRAM) [54]	Systematic review and meta-analysis	International (meta-analysis)	867 patients (339 DOACs, 528 VKAs) across 12 studies	DOACs vs. VKAs (target INR 2–3)	Systemic embolic events, overall bleeding, major bleeding, failure of thrombus resolution	No significant differences were observed in systemic embolic events (OR, 0.81), major bleeding (OR, 0.29), or failure of thrombus resolution (OR, 0.86). Overall bleeding was significantly lower with DOACs than with VKAs (OR, 0.33; *p* = 0.02).

PCI, percutaneous coronary intervention.

### 5.1 Rivaroxaban Trials

Rivaroxaban was the first DOAC to be rigorously tested in the LVT population, likely due to the once-daily dosing regimen, which simplifies the medication schedules for post-MI patients. The evidence base for rivaroxaban is currently the most robust, anchored by landmark trials that have fundamentally shaped modern guidelines.

#### 5.1.1 The RIVAWAR Trial

The RIVAWAR trial (Shah et al., 2025) [17] is the largest and most statistically powered randomized trial to date comparing a DOAC with warfarin for LVT. The study design addressed the primary criticism of earlier pilot studies: insufficient sample size and limited endpoint rigor.

The RIVAWAR trial was a single-center, prospective, open-label RCT that enrolled patients with confirmed LVT, predominantly post-anterior STEMI. Patients were randomized 2:1 to receive either rivaroxaban 20 mg daily or warfarin (target INR 2.0–3.0), both in conjunction with DAPT. The study was powered to detect differences in both thrombus resolution and clinical outcomes.

The primary efficacy endpoint was complete resolution of acute LVT assessed by echocardiography at 12 weeks (3 months). The trial demonstrated that rivaroxaban was non-inferior to warfarin for this primary composite endpoint. However, the most clinically important finding emerged from the secondary analysis of resolution kinetics. Rivaroxaban achieved significantly higher rates of complete thrombus resolution at 4 weeks (20% vs. 8%; *p* = 0.017). This “early separation” of the curves supports the hypothesis that the rapid onset of action of a direct Xa inhibitor, without the bridging gap or subtherapeutic variability associated with warfarin, leads to faster clot lysis during the period of highest embolic risk.

There was no significant difference in BARC (Bleeding Academic Research Consortium) type 3 or higher bleeding between the two arms, reassuring clinicians that the higher peak concentration associated with the once-daily DOAC dosing did not translate into excess hemorrhage in this fragile population.

#### 5.1.2 The No-LVT Trial

Before the RIVAWAR study, the No-LVT trial [18] provided the first randomized evidence challenging the VKA standard. Conducted in a population of 79 patients with anterior STEMI and echocardiographically confirmed LVT, this trial was instrumental in introducing the concept of “resolution velocity” as a therapeutic goal.

Patients were randomized to rivaroxaban (20 mg/day) or warfarin. A key strength of the No-LVT study was the aggressive imaging protocol, with resolution assessed at 1, 3, and 6 months. Moreover, the study found that rivaroxaban was superior to warfarin in achieving complete thrombus resolution:

• 1-month resolution: 71.8% in the rivaroxaban group vs. 47.5% in the warfarin group.

• 3-month resolution: 76.9% vs. 67.5%.

• 6-month resolution: by the end of the follow-up, the gap had narrowed, with 87.2% resolution in the rivaroxaban arm vs. 80.0% in the warfarin arm.

These results suggested that, while warfarin was ultimately effective for many patients, warfarin is slower to act. In a condition where the risk of stroke is highest in the first month, the drug speed becomes a safety feature. The TTR for the warfarin group was 82.3%.

#### 5.1.3 Rivaroxaban Versus Warfarin in Post-ACS Left Ventricular Thrombus Trial

Building on the momentum of No-LVT, Mansouri et al. [44] published a focused RCT specifically targeting the post-ACS population, a group often excluded from or underrepresented in broader all-comer LVT registries.

This trial aimed to validate the safety of combining rivaroxaban with DAPT, a regimen that effectively constitutes triple therapy (aspirin + P2Y12 inhibitor + anticoagulant) for 1 month. The study confirmed non-inferiority for thrombus resolution.

#### 5.1.4 The REWARF-STEMI Trial

The REWARF-STEMI trial [45] adds an important layer of validation specifically for the high-risk STEMI population. Unlike broader trials that often include patients with remote infarction or chronic cardiomyopathy, this open-label, multicenter RCT focused exclusively on patients diagnosed with LVT during the acute phase of STEMI.

A total of 50 patients with acute STEMI and confirmed LVT were randomized in the trial and set to receive either rivaroxaban (15 mg once daily) or warfarin (target INR 2.0–2.5) for 3 months. At the 3-month follow-up, complete LVT resolution was observed in 76% of the rivaroxaban arm versus 54% in the warfarin arm (*p* = 0.12). Although the trial was not powered to demonstrate statistical superiority due to the included small sample size (n = 50), the numerical trend strongly favored the DOAC arm, replicating the signal seen in the earlier No-LVT trial. Crucially, the study reinforced the safety of this strategy in the context of DAPT. There were no major bleeding events (BARC type 3–5) in the rivaroxaban arm, providing reassurance that substituting warfarin with a DOAC in the fragile post-STEMI period does not increase hemorrhagic risk.

To summarize, the RIVAWAR (2025), No-LVT, Mansouri et al. (2024) [44], and REWARF-STEMI trials collectively establish rivaroxaban as a robust alternative to warfarin for the treatment of LVT, particularly in the high-risk post-STEMI population. RIVAWAR, the largest RCT to date, demonstrated non-inferiority for the primary composite endpoint while highlighting significantly faster thrombus resolution at 4 weeks (20% vs. 8%), a finding echoed by the “resolution velocity” observed in the No-LVT trial (71.8% vs. 47.5% at 1 month). These studies suggest that the rapid onset and consistent pharmacokinetics of rivaroxaban mitigate the subtherapeutic risks inherent in the “bridging gap” of warfarin without increasing the risk of major bleeding (BARC type 3–5). Ultimately, these data support the clinical shift toward DOACs to achieve earlier clot lysis during the period of highest embolic risk.

### 5.2 Apixaban Trials

While rivaroxaban has data on treatment speed, apixaban has become the dominant DOAC in general cardiology due to the subsequent favorable bleeding profile in AF. Naturally, this advantage led to the investigation of using apixaban in patients with LVT, where a safety-first approach is often prioritized for older or frail patients.

#### 5.2.1 The AP-LVT Trial

The AP-LVT trial [24] is the primary reference for the efficacy of apixaban. This randomized, open-label trial compared apixaban (5 mg twice daily) with warfarin in patients with post-MI LVT. At 3 months, complete resolution was observed in 76% of the apixaban group compared with 80% of the warfarin group. There were no strokes in the apixaban arm, helping to allay concerns that the lower peak concentration of a twice-daily agent might be insufficient for a large ventricular thrombus.

The AP-LVT trial provided important reassurance that the standard apixaban dose, widely used for AF, is sufficient in the ventricular setting. The trial also established parity for apixaban with warfarin without the need for INR monitoring.

#### 5.2.2 Apixaban vs. Warfarin in Left Ventricular Thrombus

Alcalai et al. [2] conducted a prospective randomized clinical trial that provided more granular insight into the comparative safety of apixaban versus warfarin. This randomized pilot study enrolled 35 patients and explicitly highlighted the consistency gap between the agents. Patients in the warfarin arm achieved a mean TTR of only 60%, whereas the apixaban arm had 100% adherence to the fixed-dose regimen.

Despite the TTR limitations in the warfarin group, resolution rates were statistically similar between groups. However, the study observed a numerical trend toward fewer minor bleeding events in the apixaban arm, supporting the hypothesis that the stable pharmacokinetics of apixaban may reduce bleeding-related complications compared with the labile nature of VKAs. Notably, however, the sample size may have been insufficient to detect statistically significant differences, particularly for bleeding outcomes.

#### 5.2.3 Apixaban versus Warfarin: A Pilot Randomized Study

Isa et al. [46] reported a pilot, prospective, randomized study comparing the efficacy and safety of apixaban versus warfarin (target INR 2.0–3.0) in 27 patients with echocardiographically confirmed LVT. The primary endpoint was the percentage reduction in thrombus size or total resolution at 12 weeks, assessed by serial echocardiography. Statistical analysis was performed using analysis of covariance (ANCOVA) to evaluate differences in thrombus regression between the two treatment cohorts.

The results demonstrated no statistically significant difference in LVT resolution between the groups (*p* = 0.816). At the 12-week follow-up, the apixaban arm showed a mean thrombus size reduction of 65.1% compared with 61.5% in the warfarin arm. Safety outcomes were similar between groups, suggesting that apixaban is a non-inferior alternative to conventional VKA therapy for LVT management and may facilitate shorter hospitalizations.

To summarize, these three trials demonstrate that apixaban is a safe and effective alternative to warfarin for the management of LVT. These randomized prospective studies consistently showed no statistically significant difference in thrombus resolution rates at 3 months, with Youssef et al. [24] reporting a slightly lower rate of thrombus resolution (76% vs. 80%) and no embolic events in the apixaban arm. While warfarin therapy often lacked consistency, as reflected by a mean TTR of 60% in the Alcalai study, apixaban offered superior pharmacological stability and a trend toward fewer minor bleeding events. Collectively, these findings suggest that the fixed-dose regimen of apixaban provides sufficient anticoagulation to achieve LVT regression, while potentially improving patient adherence and reducing the need for intensive INR monitoring compared with traditional VKAs.

## 6. Observational Data

While RCTs provide the highest level of evidence for efficacy, real-world observational data offer critical insight into how treatments perform across broader, less selected populations. In the context of LVT, observational studies have played a pivotal, and at times controversial, role in shaping clinical practice. The narrative has evolved significantly, from early retrospective signals suggesting harm associated with DOACs to more recent, large-scale analyses indicating outcomes comparable to those with VKAs.

### 6.1 The RED VELVT Caution: Analysis of the 2.6-Fold Higher SSE Risk Reported in Early Retrospective Studies

For several years, the primary barrier to the adoption of DOACs for LVT was the RED VELVT study, published by Robinson et al. [16]. As the largest multicenter retrospective study at the time, the trial provided the first robust dataset that called into question the safety of off-label DOAC use for this indication.

The study analyzed 514 patients with echocardiographically diagnosed LVT across three tertiary academic centers between 2013 and 2019. Of these, 300 patients received warfarin and 185 received a DOAC, predominantly apixaban or rivaroxaban. The study was designed to assess the real-world effectiveness of these agents in preventing systemic embolization, addressing a critical gap in the absence of randomized data.

The RED VELVT trial findings were alarming to the cardiology community. The authors found that DOAC treatment was associated with a significantly higher risk of SSEs than warfarin. In the multivariable analysis, after adjusting for potential confounders, including prior SSE and thrombus mobility, DOAC use was associated with a 2.64-fold increase in SSE risk (hazard ratio (HR), 2.64; 95% confidence interval (CI), 1.28–5.43; *p* = 0.01) compared with warfarin. The divergence in event rates was profound. Survival curves showed a significant separation, particularly after the initial 3-month period, suggesting that the protective effect of DOACs may diminish over time or fail to suppress late-onset embolic events.

Interestingly, despite the difference in embolic outcomes, the study found no significant difference in the rate of thrombus resolution between the two strategies (HR, 1.08; *p* = 0.64). This disconnect suggested that echocardiographic “resolution” might not equate to elimination of embolic risk, or that DOACs were failing to suppress embolization from thrombi that appeared resolved or stable. The authors acknowledged several limitations inherent to the retrospective design. Notably, the authors lacked the TTR data for the warfarin arm and could not verify adherence in the DOAC arm.

Additionally, data presented in the supplementary materials suggest that the reported increase in SSE risk may not be fully robust. These analyses highlight several potential sources of bias, including treatment crossover during follow-up, the inclusion of patients with AF as a competing indication for anticoagulation, and heterogeneity in SSE risk by LVT etiology. However, the authors argued that the magnitude of the risk signal, a near-tripling of risk, was unlikely to be explained solely by these unmeasured variables. The study concluded with a strong cautionary note, stating that the results “challenge the assumption of DOAC equivalence” and urging caution in off-label use until randomized trials are completed. This paper effectively tempered enthusiasm for DOACs and reinforced a conservative, warfarin-first approach in guidelines for several years.

### 6.2 Large-Scale EHR Analysis (Kassab et al., 2026): Propensity-Matched Data For Parity in Real-World Outcomes

As the clinical landscape shifted and DOAC use became more widespread, larger datasets became available for analysis. Indeed, Kassab et al. [47] published a large retrospective cohort analysis in 2026 using the TriNetX research network, which aggregates de-identified electronic health records from approximately 115 million patients. This study represents the modern counter-narrative to RED VELVT, using a sample nearly five times larger and rigorous propensity score matching (PSM) to approximate a randomized trial design.

The investigators identified 2488 eligible adults with echocardiographically confirmed LVT treated between 2016 and 2022. Meanwhile, to ensure a cleaner comparison, the authors excluded patients with confounding indications for anticoagulation, such as AF, venous thromboembolism, or mechanical valves. Using 1:1 PSM, the authors created two well-balanced cohorts of 945 patients each (DOACs vs. warfarin). Unlike earlier studies in which anticoagulant choice may have been influenced by baseline characteristics (*e*.*g*., frailty) or treatment convenience, the matched cohorts in this study were similar across virtually all baseline characteristics, including age, comorbidities, and antiplatelet use.

The DOAC cohort was treated predominantly with apixaban (74%) and rivaroxaban (26%), reflecting contemporary prescribing patterns.

In contrast to the RED VELVT trial findings, Kassab et al. [47] found no evidence of DOAC inferiority. At 1 year, the composite outcome of all-cause mortality and stroke/transient ischemic attack (TIA) did not differ significantly between the groups (23.8% for DOACs vs. 26.7% for warfarin). The matched hazard ratio was 0.93 (95% CI: 0.78–1.12; *p* = 0.46), indicating statistical parity. Looking specifically at the embolic risk that drove concern in the RED VELVT trial, the 1-year risk of stroke was 13.9% in the DOAC group versus 15.1% in the warfarin group (HR 0.95; *p* = 0.67). Major bleeding rates were also comparable at 1 year (4.8% vs. 4.7%; HR 1.13; *p* = 0.58), suggesting that the switch to DOACs does not result in increased hemorrhage. Consistent with prior data, thrombus resolution rates at 6 months were similar between groups (~81%), reinforcing that both agents are effective in clot dissolution.

The Kassab et al. [47] analysis explicitly addressed the discrepancy with the earlier RED VELVT findings. Indeed, Kassab et al. [47] suggested that the higher stroke rates reported by Robinson et al. [16] may have been influenced by the inclusion of patients who switched therapies and baseline imbalances that persisted despite adjustment. Meanwhile, using a strictly matched cohort and excluding therapy switchers, the authors argued that the subsequent findings provide a more accurate estimate of the true pharmacologic efficacy of DOACs. The progression from Robinson et al. [16] to Kassab et al. [47] illustrates the maturation of the clinical evidence. The early red flag of a 2.6-fold increase in risk served an important purpose by reinforcing caution and prompting more rigorous investigation. However, the most recent large-scale data support the hypothesis that DOACs are a reasonable, safe, and effective alternative to warfarin for the management of LVT, with no excess risk of mortality or SSE in a well-matched population. Nonetheless, larger RCTs powered to detect small differences in hard primary endpoints, such as SSE and mortality, remain needed [48].

### 6.3 Daher et al. (2020): Comparative Efficacy and the Role of VKA Escalation

In a retrospective observational study conducted in France, Daher et al. [50] evaluated 59 consecutive patients with LVT detected using TTE to compare the efficacy of VKAs and DOACs. The cohort was predominantly male (83.1%) with a mean age of 62 years, and the primary etiology was ischemic cardiomyopathy (86.5%), often associated with apical akinesia. Treatment included 42 patients (71.2%) receiving VKAs (warfarin, acenocoumarol, or fluindione) and 17 (28.8%) receiving DOACs (apixaban, rivaroxaban, or dabigatran).

The study found no significant difference in thrombus resolution rates between the two groups: 70.6% in the DOAC arm versus 71.4% in the VKA arm (*p* = 0.9). Similarly, the incidence of embolic events during treatment was comparable, occurring in 11.8% of DOAC-treated patients and 9.5% of VKA-treated patients (*p* = 0.8). An important finding related to management failure was the strategy used in non-responders: five patients in the DOAC group who did not achieve thrombus resolution were switched to VKA therapy with a higher target INR of 3–4. Following this treatment intensification, all five patients (100%) achieved complete thrombus dissolution. The authors concluded that although the initial efficacy of DOACs and VKAs is similar, VKAs remain the standard of care for refractory cases because these agents can be titrated to higher therapeutic intensity by adjusting the INR.

### 6.4 Iqbal et al. (2020): Safety and Clinical Event Rates in a UK Cohort

Iqbal and colleagues [49] provided additional real-world evidence in a retrospective cohort study of 84 patients managed for LVT at a tertiary center in the United Kingdom. The study compared outcomes in 62 patients treated with VKAs and 22 treated with DOACs, predominantly rivaroxaban and apixaban, over a mean follow-up period of 3 years. As in other cohorts, the population was predominantly male (89%) with a high prevalence of ischemic heart disease (87%). The results suggested a favorable safety profile for DOACs. Clinically significant bleeding occurred exclusively in the VKA group (10% vs. 0% in the DOAC group), although this difference did not reach statistical significance (*p* = 0.13), likely due to the small sample size. In terms of efficacy, there were no statistically significant differences in stroke or SSE rates between the VKA and DOAC groups (2% vs. 0%, respectively; *p* = 0.55). Thrombus resolution on repeat imaging was observed in 76% of VKA-treated patients versus 65% of DOAC-treated patients (*p* = 0.33), suggesting comparable effectiveness in clot resolution. The authors noted a potential limitation related to imaging modality: while CMR was frequently used for diagnosis, follow-up often relied on TTE, which may have confounded the reported resolution rates. Overall, the study concluded that DOACs appear to be at least as effective and safe as VKAs for stroke prevention in this setting, offering a reasonable and more convenient alternative for patients.

## 7. Specialized Clinical Scenarios

### 7.1 LVT in Non-Ischemic Cardiomyopathy

While the vast majority of randomized data focuses on LVT in the setting of acute MI, specifically anterior STEMI, a significant minority of LVT cases occur in patients with non-ischemic cardiomyopathy (NICM). The pathophysiology of NICM differs significantly from that of the post-MI setting. In contrast, ischemic LVT is driven by a combination of endocardial tissue injury (inflammation) and stasis; thrombus formation in NICM is primarily a consequence of severe stasis caused by global hypokinesis and ventricular dilation [31,55].

Retrospective analyses have attempted to address the evidence gap in this sub-population. In the RED VELVT study, Robinson et al. [16] included a mixed cohort that contained patients with non-ischemic cardiomyopathy. However, the study was not powered to determine whether anticoagulant efficacy differed according to cardiomyopathy type. Similarly, the large-scale analysis by Kassab et al. [47] identified LVT in both ischemic and non-ischemic populations and noted that LVT poses a significant risk of embolism regardless of etiology.

More recent studies have shown that the underlying etiology of LV dysfunction can significantly influence both treatment response and outcomes in patients with LV thrombus [56]. Current clinical consensus often extrapolates management strategies from ischemic guidelines, treating NICM patients with anticoagulation for 3 to 6 months. However, unlike the post-MI setting, where the substrate (the injured apex) may heal and scar, the substrate in NICM (global dysfunction) is often chronic. Consequently, discontinuation of anticoagulation in NICM is typically guided by LVEF recovery rather than by a fixed treatment duration, unless major bleeding occurs [13]. Notably, there is insufficient evidence to determine whether anticoagulation should be continued indefinitely.

### 7.2 Triple Therapy Considerations

The management of LVT becomes substantially more complex when a patient also requires concurrent DAPT following recent coronary stenting. This combination of an oral anticoagulant (OAC), aspirin, and a P2Y12 inhibitor constitutes “triple therapy”, a regimen associated with a well-documented increase in bleeding risk. In real-world LVT cohorts, the prevalence of concomitant antiplatelet use is high. Robinson et al. [16] reported that nearly 70% of patients in the warfarin arm and 64% in the DOAC arm were receiving some form of antiplatelet therapy. Despite this intensive antithrombotic regimen, bleeding events requiring treatment cessation occurred in both groups, although the study was not designed to adjudicate bleeding as a primary endpoint. More recent data from Kassab et al. [47] provide reassurance regarding the safety of DOACs in this high-risk context. In the subsequent matched cohort, 75% of patients were receiving aspirin, and 53% were receiving P2Y12 inhibitors [47]. Despite this substantial antiplatelet therapy burden, there was no significant difference in major bleeding events between the DOAC and warfarin groups at 1 year (4.8% vs. 4.7%; matched HR: 1.13; *p* = 0.58). This similarity suggests that the elevated bleeding risk associated with triple therapy is a class effect of regimen intensity rather than a specific limitation of DOACs, thereby supporting the use of DOACs as a safer, more convenient alternative to warfarin even in post-PCI patients. Current strategies generally favor minimizing the duration of triple therapy, often to 1 week, followed by dual therapy (OAC + P2Y12 inhibitor) to mitigate hemorrhagic complications [32].

## 8. Conclusion and Future Directions

The management of LVT has undergone a paradigm shift over the past decade, transitioning from an era of empiricism and extrapolation to one increasingly guided by randomized evidence and precise imaging. As the therapeutic landscape has evolved from the rigid mandate of VKAs to the adoption of DOACs, the clinical focus has broadened.

### 8.1 Current Consensus

The publication of the 2022 AHA Scientific Statement [13] marked a watershed moment, formally endorsing DOACs as a reasonable alternative to warfarin. This consensus is not based on a single definitive trial, but rather on the accumulation of consistent signals from both RCTs and robust real-world registries.

The integration of data from trials such as No-LVT [18] and RIVAWAR [17] has established two critical precedents. First, DOACs, specifically rivaroxaban, appear to achieve thrombus resolution more rapidly than warfarin. This “resolution velocity” is mechanistically plausible, given the immediate onset of DOAC action compared with the variable induction period of VKAs. Second, the safety profile of DOACs, particularly regarding intracranial and fatal bleeding, is consistently superior to that of VKAs across AF and venous thromboembolism, and this benefit appears to extend to the LVT population.

However, a degree of caution remains necessary. Existing RCTs were primarily powered for surrogate imaging endpoints (thrombus resolution) rather than hard clinical outcomes (systemic embolization or stroke). While meta-analyses suggest non-inferiority for embolic protection, the absolute number of stroke events in these trials remains low, creating a statistical blind spot. Consequently, while DOACs are now the preferred first-line agent for many clinicians due to the associated predictable pharmacokinetics and ease of use, warfarin remains the standard for patients with specific contraindications to DOACs, such as severe renal impairment or antiphospholipid syndrome.

### 8.2 Ongoing Research

Future LVT research must address the “diagnostic gap” between TTE and CMR. As detailed in previous sections, TTE, even with contrast, fails to detect a substantial proportion of mural thrombi. Therefore, future trials must determine whether “resolution” on TTE is a sufficient endpoint or merely a source of false reassurance. Notably, ongoing and future studies, such as the Early Treatment by Rivaroxaban Versus Warfarin in ST-segment Elevation Myocardial Infarction with Left Ventricular Thrombus (EARLY-MYO-LVT) trial [57], are expected to provide further clarity. These next-generation trials are anticipated to refine our understanding in three key areas:

• Optimal duration: Whether 3 months of anticoagulation is sufficient for all patients, or whether those with persistent apical aneurysms require prolonged prophylaxis.

• The role of CMR: Whether CMR should become the mandatory gatekeeper for discontinuing anticoagulation, ensuring that “resolution” is histological rather than merely echocardiographic.

• Triple therapy de-escalation: How best to manage the intersection of antiplatelet therapy for coronary stents and anticoagulation for LVT. Future protocols will likely emphasize shorter durations of triple therapy (*e*.*g*., 1 week to 1 month) followed by dual therapy to mitigate bleeding risk without compromising ischemic protection.

### 8.3 Final Summary

Ultimately, the choice of anticoagulant should arise from shared decision-making that balances the urgency of thrombus resolution with the lifestyle and risk profile of each patient.

For the majority of patients with a new LVT post-MI, a DOAC-first strategy is now scientifically justified and clinically pragmatic. Indeed, this approach offers the distinct advantages of rapid therapeutic onset, which is crucial for stabilizing the friable thrombus, and freedom from the dietary restrictions and monitoring burdens associated with warfarin. This is particularly important for younger, active patients who may find INR monitoring disruptive.

However, a resolution-guided strategy remains paramount. Therapy should not be arbitrarily stopped at 3 months; instead, treatment should be discontinued only after imaging confirms thrombus clearance. For patients with challenging anatomy or suboptimal TTE windows, a low threshold for confirmatory CMR is advised before cessation. Meanwhile, by combining the pharmacologic precision of DOACs with rigorous serial imaging, clinicians can effectively neutralize the threat of LVT, transforming what was once a feared complication of infarction into a manageable, transient event.
